# Microbiological Identification and Susceptibility Testing Using an Automated Method in a Tertiary-Care Public Hospital in Brazil

**DOI:** 10.1017/ash.2021.117

**Published:** 2021-07-29

**Authors:** Valeria Midori, Gutoski Yuki, Patricia Rocha, Ariadne Decarli, Larissa Esteves, Laís Nascimento, Felipe Tuon, Victoria Ribeiro, Juliette Cieslinski

## Abstract

**Background:** The use of the automated system for identification and susceptibility tests can improve antimicrobial stewardship. The reduction in the time of identification of the pathogen and the correct dose of antibiotic are factors that contribute significantly to institutional programs and patient outcomes. **Objective:** We identified and evaluated the susceptibility tests of microorganisms for common pathogens through antibiograms that accounted for the minimum inhibitory concentration (MIC), in a tertiary-care public hospital in Brazil. **Methods:** This retrospective, cross-sectional study was performed to identify microbiologic profiles after the implementation of a VITEK 2 system at a tertiary-care public hospital in Curitiba, Brazil. Based on data from the medical records, patients with positive cultures of clinical samples from August to December 2017 were included in this study. The analysis included culture results, susceptibility profiles, and MICs of 5 antibiotics: amikacin, cefepime, ciprofloxacin, meropenem and vancomycin. **Results:** In total, 545 antibiograms were evaluated using VITEK 2. The following microorganisms were isolated: 345 gram-negative bacilli (63.3%), 187 gram- positive cocci (34.3%), 9 unidentified microorganisms (1.7%), and 4 yeasts (0.7%). Among the analyzed antibiograms, amikacin was tested in 371 isolates (68.1%), with an MIC of 2 mg/L being the most prevalent value, with a frequency of 224 results (41.1%). Cefepime was tested in 319 isolates (58.5%), with an MIC of 1 mg/L being the most prevalent, with a frequency of 177 results (32.5%). Ciprofloxacin was tested in 470 isolates (86.2%), with an MIC of 0.25 mg/L being the most prevalent value, with frequency of 189 results (34.7%). Meropenem was tested in 318 isolates (58.3%), with an MIC of 0.25 mg/L being the most prevalent value, with a frequency of 223 results (40.9%). Vancomycin was tested in 157 isolates (28.8%), with an MIC of 1 mg/L being the most prevalent value, with frequency of 87 results (16%). **Conclusions:** When analyzing the most frequently isolated microorganisms and their predominant sensitivity profiles in our institution, amikacin proved to be a good therapeutic option, considering the epidemiological profile, as gram-negative bacilli showed greater sensitivity. Furthermore, VITEK 2 systems provided early access to appropriate antimicrobial therapy for patients, which is a known factor for reducing bacterial resistance.

**Funding:** No

**Disclosures:** None

